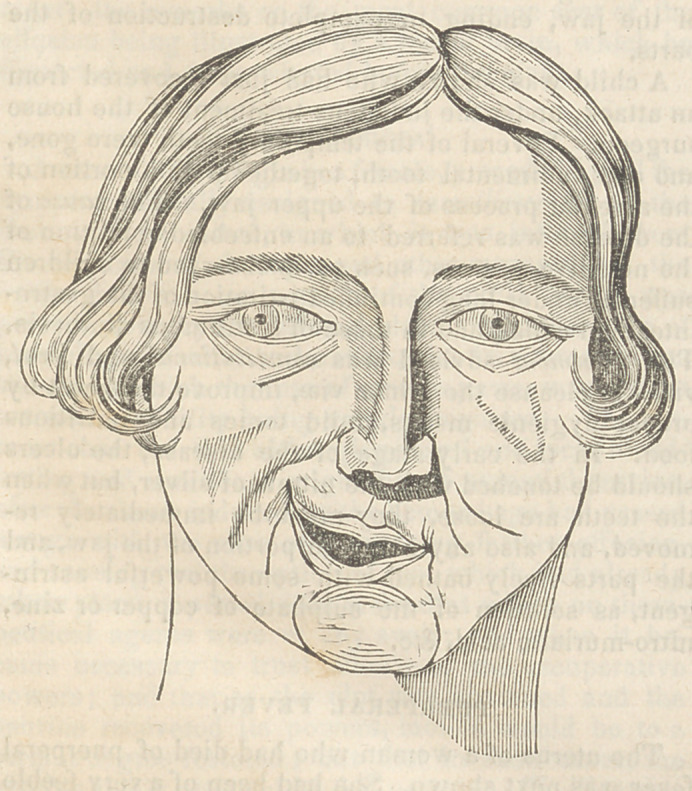# Successful Operation for Contraction of the Muscles of the Ace, Producing Extensive Deviation of the Right Angle of the Mouth

**Published:** 1844-01-13

**Authors:** 


### THE MEDICAL EXAMINER,

### And Record of Medical Science.

Vol. VII.] PHILADELPHIA, SATURDAY, JANUARY 13, 1844. [No. 1.

### SUCCESSFUL OPERATION FOR CONTRACTION OF THE MUSCLES OF THE FACE,

*Producing extensive deviation of the right angle of the Mouth.*

BY PROFESSOR PANCOAST.

Joseph W——, a lad 19 years of age, an apprentice of Mr. John Struthers, of Philadelphia, has been affected, since his infancy, with a contraction of the muscles of the face, represented in the accompanying cut; otherwise he is healthy and active. The cause of the disease is not positively known; the distortion of the face was, however, first noticed by his parents after his recovery from a severe attack of measles. The deformity, according to the report of his master, has been slowly but steadily on the increase. When his mouth is at rest, the left commissure of the lips is placed nearly under the corresponding nostril; the right commissure being carried outwards and upwards upon the cheek. In attempting to smile or speak, or simply open the mouth, the deformity becomes greater, the right commissure being carried farther outwards, and at the same time either upwards or downwards, according to the muscles of the side put in action, and raising concentric ridges upon the cheek. On the left side the cheek is flattened, and the patient is almost entirely without voluntary control over the muscles. On attempting to draw the mouth to the left side, some tremulous motion only is produced in the corner, while the muscles of the right side are thrown, by the effort, into strong contraction.

In a case of this description it would be difficult, from mere inspection of the parts, to say whether the deformity was owing to the paralysis of one set of muscles, or to the excessive spastic action of those of the other side. Some unusual prominence obvious in the left eyeball, without other probable cause than a weakened action of the recti muscles, inclined me, however, to the latter opinion.

Under either view of the case, the patient could hardly fail to be benefitted by an operation, which, without producing any cutaneous wound, should so weaken the action of the muscles of the right side as to give those of the left a chance of gaining a relative increase of strength, and enable them to bring the mouth into its proper position.

June 9, 1842. I performed the following operation in the case at the clinic of the Jefferson Medical College. The patient was seated in a chair. On introducing my finger into the mouth, and causing him to attempt a smile, I found a roundish, rigid hardening of the muscles in three different directions—that of the buccinator—that of the zygomatici—and that of the depressor anguli oris. The orbicularis seemed also at fault, as it sunk the corner of the mouth inwards. Two subcutaneous incisions with a long and very narrow bistoury, straight on the edge, were made to divide these muscles. The knife was entered on the side of the mucous membrane, for the purpose of preventing the slight cicatrix, which might follow the puncture, from being visible. For the first incision, the knife was entered just above and in front of the entrance of the parotid duct, and pushed cautiously along the cutaneous surface of the mucous membrane in a direction parallel with the alveolar processes of the upper jaw, and for the extent of about two inches; the edge of the blade was then turned in front, and all the parts between the mucous membrane and skin divided as it was withdrawn. The zygomatic muscles gave way with a snap, and the buccinator was cut through the greater part of its origin from the upper jaw bone. The upper lip was pushed outwards with the thumb and finger, and the knife, turned forwards as upon a pivot, divided the orbicularis oris through to the epithelium of the lip, without increasing the size of the puncture at the place of its entry. Four muscles were thus divided at one incision, as well as a portion of the fibres of the levator muscles. Considerable hemorrhage followed the withdrawal of the knife, though precaution had been taken to compress the facial artery. The blood filling up the line of the cut, gave an increased fulness to the cheek; the bleeding quickly stopped of itself, but little taking place externally.

The knife was then introduced, in like manner, from the inner surface of the lower lip just within the commissure, and carried obliquely downwards towards the angle of the jaw, and made to divide, as it was withdrawn, all the parts between the skin and mucous membrane up to the covering of the lip, consisting of the lower edge of the buccinator, the hard and rounded depressor anguli oris, and the lower disk of the orbicularis—the movement of the point of the knife being obvious below the skin in its whole course as it was withdrawn. But little bleeding followed this incision. The mouth, as was apparent to all the spectators, became immediately straight; nearly all power of motion over the right corner of the mouth was lost, while the patient regained considerable control over the left. A compress was secured with a nodose bandage over the facial artery. With a small silver hook in the left commissure, attached to a piece of ribbon, the mouth was drawn as far as possible to the left side, for the purpose of widening the subcutaneous incisions made on the right side, and allowing them to fill up with a thick stratum of lymph, which, after the closure of the wound, is to insulate the divided portions of the muscles. The first incision only was much painful; and the patient suffered afterward so little as to be unwilling to confine himself within doors.

June 10. The patient feels no other inconvenience from the operation than some slight soreness of the cheeks when touched. Some fulness of the cheek is still apparent, and a hardened rounded line of effused blood and lymph can be felt along the track of the incision. The nodose bandage was removed. The astringent application and the use of the hook still continued. The wounds at the places of puncture were scarcely obvious.

June 12. Finding the hook becoming painful, it was yesterday removed. The swelling and soreness of the cheek has almost entirely disappeared. On causing the patient to put into play the muscles of the right side of the face, it was found that none acted on the mouth, to produce deformity, but the middle undivided part of the buccinator, and the depressor labii inferioris of that side. A bistoury was introduced, as before, under the mucous membrane, and the middle part of the buccinator divided transversely, by a subcutaneous cut, about three-quarters of an inch from the commissure. The excessive traction of the corner of the mouth outwards at once ceased. The depressor still jerked the lip downward; but the division of it was deferred till the tenderness resulting from the former operations had ceased.

June 13. More pain and soreness has followed the last comparatively small incision than attended the two former ones; serving to show the propriety of making all the necessary subcutaneous incisions in such cases, when possible, at one sitting, or waiting till every shade of inflammatory action had subsided before making a second cut. The patient, however, walks about, and is able to resume his business.

July 1. Every trace of swelling and soreness is removed from the face. The patient, though not entirely cured, is very greatly improved in feature and expression. The mouth, when at rest, is perfectly straight. The muscles of the left side of the face have not regained much power, with the exception of the zygomatic, which act with considerable force. The muscles on the right side produce little more, when put in contraction, than their natural effect. His articulation is more distinct than before the operation; and the only obvious defect now existing, is that produced by the depressor labii inferioris.

## Figures and Tables

**Figure f1:**